# Biologics in allergology and clinical immunology: Update on therapies for atopic diseases, urticaria, and angioedema and on safety aspects focusing on hypersensitivity reactions 

**DOI:** 10.5414/ALX02533E

**Published:** 2024-11-14

**Authors:** Uta Jappe, Karl-Christian Bergmann, Folke Brinkmann, Valentina Faihs, Askin Gülsen, Ludger Klimek, Harald Renz, Sebastian Seurig, Christian Taube, Stephan Traidl, Regina Treudler, Martin Wagenmann, Thomas Werfel, Margitta Worm, Thorsten Zuberbier

**Affiliations:** 1 Division Clinical and Molecular Allergology, Research Center Borstel, Leibniz Lung Center, Airway Research Center North (ARCN), Member of the German Center for Lung Research (DZL), Borstel,; 2 Interdisciplinary Allergy Outpatient Clinic, Department of Pneumology, University Medical Center Schleswig-Holstein, Campus Lübeck, University of Lübeck,; 3 Institute of Allergology, Charité Universitätsmedizin Berlin und Fraunhofer Institute for Translational Medicine and Pharmacology ITMP, Allergology and Immunology, Berlin,; 4 Division of Pediatric Pulmonology and Allergology, University Children’s Hospital, German Center for Lung Research (ARCN, DZL), Lübeck,; 5 Department of Dermatology and Allergy Biederstein, Klinikum rechts der Isar, Technical University of Munich,; 6 Division of Cardiology, Pulmonary Diseases, Vascular Medicine, University Hospital Duesseldorf,; 7 Center for Rhinology and Allergology, Wiesbaden,; 8 Institute of Laboratory Medicine, Universities of Giessen and Marburg Lung Center (UGMLC), Philipps Universität Marburg, Member of the German Center for Lung Research (DZL) Marburg,; 9 Interdisciplinary Allergy Center Nuremberg (NIZA), Department of Internal Medicine 3, Pneumology, Nuremberg Hospital, Nuremberg,; 10 Department of Pulmonary Medicine, University Hospital Essen-Ruhrlandklinik, Essen,; 11 Department of Dermatology and Allergy, Hannover Medical School, Hannover,; 12 Institute of Allergology IFA, Charité Universitätsmedizin corporate member of Freie Universität Berlin and Humboldt-Universität zu Berlin, Campus Benjamin Franklin, Berlin,; 13 Department of Otorhinolaryngology, Düsseldorf University Hospital, Heinrich Heine University, Düsseldorf,; 14 Division of Allergy and Immunology, Department of Dermatology and Allergology, Charité-Universitätsmedizin Berlin, Berlin, Germany

**Keywords:** anti-drug antibodies, biological allergy, children, eosinophilic oesophagitis (EoE), food allergy, hypersensitivity reactions, off-label use, pregnancy, COVID 19, vaccination

## Abstract

The development of targeted therapies for atopic diseases, urticaria, and angioedema with biologics is progressing rapidly: New “targets” of clinical-therapeutic relevance have been identified, the corresponding targeted antibodies developed, tested in clinical trials, and approved for therapy. These include the anti-IgE antibody omalizumab (also effective and approved for the treatment of urticaria), the anti-IL-4/13 receptor-specific antibody dupilumab, the two anti-IL-13 antibodies lebrikizumab and tralokinumab, the anti-TSLP antibody tezepelumab, the two anti-IL-5 antibodies mepolizumab and reslizumab, and the anti-IL5 receptor-specific antibody benralizumab for the treatment of atopic diseases. For the treatment of hereditary angioedema, C1 inhibitor and the antibody lanadelumab (directed against kallikrein) have also long been approved as biologics in addition to low-molecular substances. Other therapeutic antibodies are in various stages of development. Furthermore, the range of indications for some very effective biologics has been successfully expanded to include additional diseases. In this context, the first results on biologic therapy of food allergy and eosinophilic esophagitis are interesting. Biologics that address different target structures are also increasingly being administered in combination, either simultaneously or sequentially, in order to achieve optimal efficacy. A developing area is the use of biologics in children and the observation of immunological and non-immunological side effects. In some cases, new unexpected side effects and hypersensitivity reactions have emerged, which in turn raise pathomechanistic questions, such as conjunctivitis with dupilumab therapy, which only appears to occur in the treatment of atopic dermatitis but not in the treatment of other atopic diseases. In dermatology, paradoxical reactions have been described under therapy with some biologics. And immune reactions of type alpha to epsilon to biologics (hypersensitivity reactions) continue to be a clinically relevant problem, whereby the selection of an alternative therapeutic agent is a challenge and the diagnostics that support this have not yet been sufficiently incorporated into routine work.

## Introduction 

The intensive work on the pathomechanistic elucidation of inflammation and oncological processes with the aim of finding biomarkers for improved diagnostics and specific targets for therapies (target treatments) as well as preventive measures has already made important contributions to the individual treatment of patients in recent years. Since the first edition of our work on biologics in atopic diseases [1], there have been important further developments, which are discussed below on an organ-associated basis. 

Biologics are therapeutically used substances that imitate or directly and specifically address key players of the human immune system or organism and can thus modulate pathological processes. Depending on the mechanism, this is achieved in different ways. 

They are essentially active substances from the following groups: monoclonal antibodies (mAb), cytokines, and fusion proteins. Their effect is triggered by binding specifically to receptors (activating or inhibiting them) and complexing active molecules in order to inhibit them (cytokine and antibody inhibitors). Effective cytokine inhibitors are antibodies directed against IL-5, the epithelial cytokine thymic stromal lymphopoietin (TSLP), and IL-13, as well as those directed against the corresponding receptors or structural parts thereof, such as against the IL-4 receptor α-chain or the α-chain of the human IL-5 receptor, and block receptor-interleukin binding. 

Increasingly rare are the mAb chimeras. Chimeric means that they are constructed from human and mouse components and therefore have a relatively high immunogenicity/allergenicity for humans (< 50 – 75% human). For this reason, the focus is now on the production of humanized or human mAb. 

The structural combination of soluble proteins and antibody fragments (e.g., IgG1, Fc part) can imitate a ligand or a receptor as a fusion protein that has a desired strong affinity to the target structure. 

Although the design and production of therapeutics specifically address the body’s own key structures and increasingly resemble human molecules in their molecular structure, it cannot be assumed that their effect is fully understood or that their therapeutic impact is without side effects or undesirable immune reactions such as hypersensitivity or hypersensitivity reactions, induction of autoimmune diseases and immunodeficiency. For some, additional non-immunological side effects are known (e.g., for dupilumab, dry eyes and conjunctivitis or even paradoxical reactions, see below). 

Both an advantage and a challenge is the development of biologics to treat the various atopic diseases and address the correspondingly different organ systems: an advantage because they are partly based on a common pathogenesis, a challenge because still no single biologic is able to improve all atopic diseases at the same time. 

More and more, however, the development of biologics and the corresponding successes in patients are helping to better and better characterize the specifics of atopic diseases and their subgroups so that the therapeutic approach and pathomechanistic clarification go hand in hand, so to speak. 

## Biological therapy of bronchial asthma 

The first biologic approved for this indication was omalizumab in 2005. Since then, other biologics have been used to treat defined phenotypes of severe allergic asthma: These include antibodies that block IL-5 (mepolizumab, reslizumab), the IL-5 receptor (benralizumab), or the IL-4 receptor α-chain (dupilumab) [1, 2]. Although the indication for prescribing biologics is severe asthma, there is no single valid definition of this phenotype. Instead, different approaches have been published to characterize the patient group “severe asthma”. The key factor is the presence of uncontrolled asthma despite high-dose inhaled anti-inflammatory therapy (inhaled corticosteroids (ICS)) in combination with another “controller” (e.g., long-acting beta-2 sympathomimetics). Analyses of insurance and health insurance data indicate that this definition only applies to ~ 3 – 4% of patients with asthma [1, 3, 4]. Questionnaires (Asthma Control Test or Asthma Control Questionnaire), the documentation of acute exacerbations, the need for inpatient treatment, and impaired lung function are used to objectify the diagnosis of “uncontrolled asthma”. In this context, patients with “difficult-to-treat” asthma must be differentiated from patients with “severe” asthma [1, 5]. 

For the majority of patients with uncontrolled asthma despite high-dose ICS therapy, inadequate medication intake (e.g., inadequate inhalation technique, lack of adherence), previously undiagnosed or untreated comorbidities such as sleep apnea, obesity, reflux, chronic rhinosinusitis, or the persistence of relevant trigger factors such as allergen sources in the environment are the cause of poor symptom control. These factors must therefore first be eliminated and disease management optimized. If this is not successful, the patient has severe asthma. For these patients, the indication for treatment with a biologic should then be examined [1]. The recommendation of national and international guidelines is that biologic therapy should definitely be preferred to systemic corticosteroid treatment [1, 6, 7], as prolonged and repetitive systemic cortisone therapy for asthma is associated with the known undesirable side effects [1, 4, 8]. In order to identify those patients who would benefit from biologic therapy among the various asthma phenotypes with their different immunological characteristics, both the clinical and immunological criteria must be recorded very carefully. The phenotypes of severe allergic asthma, asthma with eosinophilic inflammatory reaction, and asthma with type 2 inflammation, some of which have considerable overlaps, can already be treated with monoclonal antibodies [1]. Omalizumab has been authorized for the treatment of severe allergic asthma since 2005. It can contribute to a reduction in the frequency of exacerbations, an improvement in symptoms and quality of life as well as lung function. In addition, the need for systemic steroid therapy can be reduced [1, 9]. Omalizumab is also effective regardless of the type of inflammation: a reduction in acute exacerbations has been demonstrated both in patients with and without eosinophilic inflammation [1, 10]. In practice, however, it should be noted that the use of omalizumab is not expected to reduce eosinophils in the blood. In individual cases, this can lead to a good improvement in lung function being achieved with omalizumab in severe allergic asthma, but further optimization can only be achieved with the additional use of an anti-IL 5 biologic [11]. As omalizumab has been used since 2005, the longest experience (long-term use) is also available with this biologic. Retrospective data from continuous use over 5 – 10 years in one center document that the biologic shows no loss of efficacy even after years of use and that there is no change in its good tolerability even after prolonged use [1, 12]. Three therapeutic antibodies have been approved for the treatment of severe asthma with eosinophilic inflammation: mepolizumab and reslizumab (anti-IL-5 antibodies) as well as benralizumab (antibody against the α-chain of the human IL-5 receptor (IL-5Rα)). Mepolizumab significantly reduces exacerbations, improves asthma control, and also improves FEV_1_ in patients with an elevated eosinophil count in the peripheral blood [1, 13]. In patients with severe asthma and eosinophilia, benralizumab therapy [1, 14] also leads to a reduction in exacerbations, an improvement in symptoms, an increase in quality of life, and a slight improvement in lung function [1, 15]. These therapeutic effects of both biologics are of particular importance in the case of steroid-requiring asthma, as controlled studies have shown that the use of anti-IL-5 or anti-IL-5Rα antibodies enables a reduction or complete discontinuation of systemic corticosteroids [1, 13, 16]. Despite the reduced steroid dose, there were fewer exacerbations in the treated groups. Due to the massive side effects of systemic steroid therapy, these results are of considerable relevance. Dupilumab is also authorized for the treatment of severe asthma. It binds to the α chain of the interleukin-4 receptor (IL-4Rα) and thus inhibits the binding of IL-4 and IL-13 to the respective receptor. Dupilumab has also been shown to reduce exacerbations, improve quality of life and lung function in patients with uncontrolled asthma and evidence of eosinophilic inflammation or elevated levels of nitric oxide (FeNO) in exhaled air [1, 17]. Dupilumab enabled a reduction or complete discontinuation of systemic corticosteroids in patients who were treated with systemic steroids on a long-term basis [1, 17]. Omalizumab, mepolizumab, benralizumab, and dupilumab are now approved for self-administration at home, but this represents a risk that should not be underestimated due to the possibility that anaphylactic reactions to biologics may develop even after months of successful therapy [1, 18]. Another therapeutic option was the introduction of tezepelumab. The monoclonal antibody inhibits TSLP. TSLP is an epithelial cytokine that plays a crucial role in asthma pathogenesis as it is released following epithelial damage or activation of immune cells. The higher the blood level of TSLP, the higher the rate of exacerbations [19]. Tezepelumab is indicated as an add-on maintenance therapy in adults and adolescents aged 12 years and older with severe asthma that is inadequately controlled despite high-dose inhaled corticosteroids and another drug for maintenance therapy. In contrast to the other biologics, there are no requirements regarding the level of eosinophils in the blood, IgE or cutaneous sensitization, FeNO or lung function parameters, which minimizes the diagnostic requirements. There is no doubt, however, that it is useful and, in the absence of an effect, essential to determine these parameters in order to achieve phenotyping. The rationale for dispensing with the determination of eosinophils and FeNO lies in the efficacy of tezepelumab even with eosinophils of < 150/µL and a normal FeNO – although higher efficacy with eosinophils > 150/µL has been documented in phase III studies [20]. Biologic therapy for severe asthma should be started by physicians experienced in this disease and the efficacy evaluated after 4 – 6 months. If there is no clear response, the review phase can be extended to 12 months [1]. The following applies to the remaining concomitant medication: After starting biologics treatment, the previous inhaled and oral asthma therapy should be maintained for at least 4 weeks and only then reduced, if necessary, under close monitoring of asthma control [1]. Biologics represent an add-on therapy and are not yet authorized for monotherapy. However, the use of biologics often leads to such an improvement in lung function, Asthma Control Test (ACT) and symptoms that the patients concerned can completely dispense with the further use of inhaled steroids and long-acting beta-mimetics – which they do of their own accord [1]. Without there being a national or international recommendation for these situations, an extension of the injection intervals can be considered in these cases [1]. For omalizumab, it has already been described that once a controlled stage has been reached, it is possible to significantly extend the injection intervals [21], while a reduction or discontinuation of the biologic usually led to renewed deterioration [1]. 

In another study [22], the possibility of extending the dosing intervals of mepolizumab in patients with severe eosinophilic asthma after achieving good control was also investigated. ACT and lung function values improved significantly after starting therapy with mepolizumab at regular 4-weekly injection intervals. After extending the dosing intervals, both ACT and lung function remained at a stable level for 1 year, and the interval extensions had no effect on the use of systemic oral corticosteroids (OCS). In patients with completely or well-controlled eosinophilic asthma treated with mepolizumab, extending the dosing intervals between injections to up to 8 weeks has the potential to avoid unnecessary injections and save costs for the healthcare system. This procedure also appears possible for other biologics in individual cases without contradicting the previous indication description. 

Still formally unresolved is the question of how the treating physician should behave if the patient who feels that their asthma is almost or completely controlled refrains from taking any or almost any further asthma medication. The doctor has informed the patient that the biologic is an add-on therapy, and that it is assumed that the patient will continue to use at least ICS in addition to the biologic – after oral steroids have possibly already been discontinued completely to the delight of the patient and the doctor. 

It remains the self-evident right of every patient to use only those medications that they themselves accept. There is no compulsion to take medication and, from a legal point of view, the patient can refuse any therapy. 

However, it remains the task of the doctor caring for the patient and prescribing the biological agent, to inform the patient and to recommend and prescribe the ICS, albeit at a reduced dose. The prescriber should always document the prescription of the ICS as a medication to be used regularly and the verbal instruction to use it – even if they have the impression or feeling that their explanatory words may not be followed. 

In the same way, prescribers of all biologics for the indication severe asthma should check the indication of the prescription every 4 – 6 months and document this in writing. The number of exacerbations, the possible dose of OCS, the ACT value, and the development of lung function are important parameters that can be used for an assessment during the course of treatment with the biologic. The biologic should be assessed in dialogue with the patients – but should not just follow their wishes. 

## Biologics for the treatment of atopic dermatitis 

Dupilumab, a recombinant human IgG4 monoclonal antibody, was the first biologic approved for the treatment of moderate to severe atopic dermatitis in 2017. This antibody specifically targets the common IL-4Rα subunit of type 1 and type 2 IL-4 receptors, thereby blocking both IL-4 and IL-13. The approval was based on two placebo-controlled phase III studies and a long-term study over 1 year [23, 24]. Approval was first extended to children aged 12 years and older in the autumn of 2019, based on the results of a successful placebo-controlled study in this age group [25]. Subsequently, approval was granted for children aged 6 years and older. In 2023, this approval was further extended to include infants as young as 6 months, based on the results of respective placebo-controlled, randomized studies [26, 27]. The side effect profile of dupilumab primarily includes the risk of conjunctivitis and irritation at the injection site. Clinical studies report a variable incidence of conjunctivitis ranging from 8.6 to 22.1%. This incidence is slightly lower compared to data from clinical registries in Germany, France, Italy, and the Netherlands, where conjunctivitis prevalence ranges from 13.3 to 38.2%, depending on the time of data collection [28, 29, 30, 31, 32, 33, 34]. Increasing evidence suggests that dupilumab is only partially effective in head-neck dermatitis and that dupilumab-associated facial erythema can also occur, sometimes persisting for longer periods of time. The SCRATCH registry, documenting a Danish national cohort of 347 adult atopic dermatitis patients treated with dupilumab, shows a high drug survival rate (90% after 52 weeks, 86% after 104 weeks) [35]. However, the proportion of patients with atopic dermatitis in the head and neck region remained almost unchanged from initially 76 to 68% at week 104. A beneficial side effect noted in clinical studies is protection against severe Herpes simplex virus (HSV) infections (eczema herpeticum) by dupilumab. Recent molecular studies have shown that the treatment enhances defense against HSVs, providing an additional advantage for the subgroup of over 10% of patients with recurrent HSV infections [36]. 

Recently, single-cell RNA sequencing revealed the presence of a type 2 inflammation pattern in prurigo nodularis [37]. This finding underscores the significance of the 2023 approval extension for dupilumab, which followed two randomized, placebo-controlled phase III trials demonstrating the efficacy of dupilumab in treating prurigo nodularis [38]. In June 2021, another human monoclonal antibody, tralokinumab, was approved for the treatment of patients with moderate to severe atopic dermatitis. It is directed against the type 2 cytokine IL-13 and blocks the binding of IL-13 to both the IL-4/IL-13 receptor (IL-13Rα1) and the so-called decoy receptor (IL13Rα2). In the phase III ECZTRA1 and ECZTRA2 studies with 601 and 591 participants, 25.0% and 33.2% of patients achieved an EASI-75 response after 16 weeks without additional topical anti-inflammatory therapy [39]. The initial dose was 600 mg subcutaneously, followed by a maintenance dose of 300 mg every 2 weeks. In the clinical studies, patients who achieved an EASI-75 improvement or complete or almost complete remission of atopic dermatitis were re-randomized after 16 weeks. 

These patients either continued to receive tralokinumab every 2 weeks, were switched to a 4-week administration, or received a placebo. After a total of 36 weeks, 59.6 and 55.8% of patients continuing biweekly treatment achieved the EASI-75 endpoint, compared to 49.1 and 51.4% of patients who were switched to 4-weekly dosing. These data on the potential extension of the dosing interval were included in the marketing authorization, allowing a dose reduction in patients with a good to very good treatment response after 16 weeks. Following a phase III study, the authorization was extended to adolescents aged 12 and over [40]. Based on the study data (n = 2,855), the frequency of conjunctivitis could be slightly lower than with dupilumab at 7.5%, although unfortunately no directly comparable study is available. The benefit of switching from dupilumab to tralokinumab due to ocular side effects has so far only been described in case series, indicating a need for larger sample sizes to make a valid conclusion [41]. 

In 2023, lebrikizumab, a monoclonal antibody also targeting IL-13, received approval. Unlike tralokinumab, lebrikizumab does not inhibit the binding of IL-13 to both IL-13 receptors (IL-13Rα1 IL13Rα2), but only the binding to IL-13Rα1. In the phase III studies ADvocate1 and ADvocate2, a total of 564 and 427 patients, respectively, were examined over a period of 52 weeks, including a 16-week induction phase and a 36-week maintenance phase [42]. The initial dose of 500 mg at the beginning was followed by 250 mg every 2 weeks or the placebo dose. In the lebrikizumab group, 43.1 and 33.2%, respectively, of patients achieved the primary goal of an Investigator‘s Global Assessment (IGA) score of 0 or 1 with a reduction of at least 2 points from baseline (12.7 and 10.8% in the placebo group), and 58.8 and 52.1% achieved an EASI-75 response (16.2 and 18.1% in the placebo group). In the maintenance phase, the responders (EASI-75 response or IGA 0/1) were again randomized into 3 groups and received lebrikizumab or placebo every 2 weeks or every 4 weeks. Similar results were seen for the 2-week and 4-week doses [43]. This led to an approval, which is summarized as follows in the information for healthcare professionals: “In patients without a clinical response after 16 weeks, treatment should be discontinued, whereas those with a partial response may experience a potential improvement up to week 24 with continued two-week treatment; once a response is achieved, a monthly maintenance dose of 250 mg lebrikizumab is recommended”. 

To document the indication for systemic therapy of atopic dermatitis with biologics or other substances such as JAK inhibitors or cyclosporine, age-adapted checklists were included in the current AWMF S3 guideline on atopic dermatitis. These checklists contain objective, subjective, and therapy-associated criteria [44, 45]. Once the indication for treatment with a biologic has been established, further consideration is required as to which biologic should be selected. Biologics have demonstrated a good safety profile in both children and the elderly [46]. Recently, a review article summarized various factors that can influence the decision when choosing a systemic therapeutic [47]. In addition to the patient‘s age, existing comorbidity and other approved indications (asthma, chronic rhinosinusitis with nasal polyps (CRSwNP)), side effect profiles, disease manifestations (including the presence of head-neck dermatitis), patient wishes, injection intervals, and personal experience can also play a role. Unfortunately, molecular markers that would enable the personalized selection of a biologic are not yet available [48]. 

## Biologics for the treatment of chronic rhinosinusitis with nasal polyps 

Since 2019, biologics have been approved as add-on therapy to intranasal steroids for the treatment of severe CRSwNP in Germany. Three biologics with different target molecules are currently approved (marketing authorization): dupilumab – target molecule: IL-4Rα; omalizumab – target molecule: IgE; mepolizumab – target molecule: IL-5. The clinical use of these drugs has led to significant improvements in the treatment of patients with severe CRSwNP, particularly those with recurrent polyps following one or more previous sinus surgeries. This new treatment option has led to significant changes in clinical paradigms – in particular a reduction in recurrent surgery and at least the hope that the use of oral glucocorticosteroids, which is associated with side effects, can also be reduced as a result. 

The texts of the marketing authorization and the indication criteria are very similar for all three biologics (dupilumab, omalizumab, and mepolizumab), and all three agents led to statistically significant and clinically relevant improvements in quality of life, nasal obstruction, and a reduction in polyp size in phase III studies. To date, there are no data that would allow the selection of the optimal biologic for the individual patient on the basis of clinical parameters or laboratory values. However, a number of meta-analytical indirect comparative studies on the effect of the various biologics in severe chronic rhinosinusitis have now been published. These studies all point in the same direction. Not only do they prove the overall efficacy of the biologics in this indication, but they also indicate a greater efficacy of dupilumab compared to mepolizumab and omalizumab. 

In a publication from 2023, which is characterized by very strict criteria with regard to the selection and quality of the original studies included [49], the most important clinical results of a total of 2,021 patients with CRSwNP who had participated in 10 randomized clinical trials with a treatment duration of at least 12 weeks with the biologics benralizumab, dupilumab, mepolizumab, and omalizumab were analyzed. Subgroup analyses were performed for all clinical parameters. These showed the strongest effects for dupilumab compared to mepolizumab, omalizumab, and benralizumab for the reduction in nasal polyp scores, CT scores, nasal flow values (PNIF), olfactory test results and also for disease-specific quality of life (SNOT-22), symptom scores for olfaction and nasal obstruction. This analysis underpins the overall dominant efficacy of dupilumab in CRSwNP, with the differences in the subgroups only just exceeding the threshold of statistical significance. 

However, the authors point out of course that they only conducted indirect efficacy comparisons, that the inclusion criteria in the studies were heterogeneous, and that only direct head-to-head studies can further improve the evidence. Interestingly, such a head-to-head study for the comparison between dupilumab and omalizumab has already been started (www.ClinicalTrials.gov). 

Recommendations and guidelines on the indication and progression assessment of biologics in CRSwNP have been updated in recent years and adapted to the latest findings from a large number of new studies. Of particular relevance in this context are the recommendations of the EUFOREA/EPOS group, the update of the chapter on biologic therapy of the German S2k guideline on rhinosinusitis [50], and the recommendations of the Ärzteverband Deutscher Allergologen e.V. (AeDA) and the German Society of Oto-Rhino-Laryngology, Head and Neck Surgery (DGHNO-KHC) [51], which focus on the German healthcare system. 

The German guideline provides clinically helpful specifications for the indication of biologic therapy and its documentation. In the latest version of the EPOS/EUFOREA recommendations [52], criteria for the indication of biologic therapy were adjusted in detail compared to the previous version. Furthermore, it is recommended here that at least 3 out of 5 listed criteria should be fulfilled in order to justify the prescription of biologics. In order to recognize an underlying type 2 inflammation (one of the criteria), the threshold value for blood eosinophilia was reduced from 250 to 150 Eos/µL which is in line with the pneumological literature. In addition, the need for patient participation was explicitly included in the text. Their preference for either a surgical or drug therapy concept should be taken into account in the decision. 

Probably even more important clinically were the changes to the criteria for assessing the success of biologic therapy, which are to be carried out after 6 and 12 months and which have also been adapted for the German healthcare system [53, 54]. Furthermore, results reported by both physicians and patients are taken into account for the assessment – including nasal endoscopy and the assessment of disease-specific quality of life. The response is assessed in three categories (poor – good – excellent) in order to decide whether to continue, modify, or discontinue biologic therapy. 

In the meantime, a large number of real-life studies on biologics in CRSwNP have been published, which overall confirm the excellent efficacy of this therapy. The largest and methodologically best of the studies to date was published last year by de Corso et al. [55]. In this multicenter phase IV observational study, the efficacy and safety of dupilumab was investigated in 648 patients with severe uncontrolled CRSwNP in the first year of treatment. The evaluation showed statistically significant improvements for all parameters examined and a very high response rate of the therapy. Only 3.2% of patients (20/648) responded poorly or not at all to treatment. Similar to previous real-life studies, the observed extent of improvement with dupilumab therapy was rather higher than in the marketing authorization studies. The median nasal polyp score improved from 6 at baseline to 1 after 12 months. The quality of life measured with the SNOT-22 increased from 58 (median) at baseline to 11 (median) after 12 months. For the other two approved biologics, only limited data from real-life studies are available to date. 

These overall very positive data and experiences with biologic therapy for CRSwNP have led to two interlinked lines of discussion. The good results of biologic therapy in CRSwNP indicate that not only disease control, but also remission in many cases could be a realistic goal. EPOS/EUFOREA has therefore recently published proposals for the definition of disease control and remission in CRSwNP [56]. Here, “control” is defined as the absence of symptoms in the last month (even during ongoing therapy). For “remission”, not only symptoms but also endoscopic evidence of active disease should be absent in the last year. For patients who achieve these goals during biologic therapy, the obvious question for them is whether treatment should be de-escalated. This could be achieved by extending the dosing intervals. Van der Lans et al. [57] showed in a long-term study of high methodological quality that it is possible to gradually extend the dosing intervals of dupilumab in the majority of patients without any loss of efficacy. 

However, implementation in everyday clinical practice appears premature, as this procedure should be reviewed and confirmed in independent cohorts. It is also important to note that in the current situation, dosing interval extensions are an off-label treatment with potential legal and financial consequences. Despite many attempts, it has not yet been possible to identify biomarkers that could optimize the selection of patients for biologic therapy in CRSwNP in general or even for the selection of specific treatment antibodies for the individual patient. However, this shortcoming should be set against the fact that the response rates are very high, taking into account the currently recommended clinical parameters. 

In the future, it will be interesting to see whether other biologics currently being investigated in clinical trials will overcome the hurdle of marketing authorization and whether the good clinical success of biologic therapy will possibly lead to an expansion of its indications in chronic rhinosinusitis. 

## Biologic therapy for food allergy 

For the treatment of IgE-mediated food allergy as a potentially life-threatening disease, no biologic has yet been approved in Europe [58]. With regard to the mechanism of action of omalizumab, a positive effect can be hypothesized due to the pathogenesis of food allergy, which is confirmed by numerous case series and individual controlled prospective studies with a limited number of cases, mostly in children, whereby omalizumab is effective as monotherapy or in combination with oral immunotherapy (OIT) [59]. Omalizumab administered as monotherapy over several months can increase the tolerance threshold of the symptom-triggering food: for peanut (for which there are the most case series [60, 61]), for cow’s milk, and hen’s egg [1, 62, 63]. 

A study in children with simultaneous allergy to multiple foods on the efficacy of anti-IgE treatment showed that the group treated with omalizumab was significantly more likely to reach > 2 g protein of the culprit allergen source in food challenge in at least more than two of the food allergies compared to placebo [1, 64]. 

Also a very recent placebo-controlled prospective multicenter study on 177 children and adolescents with allergy to peanut and at least two other foods showed superiority of omalizumab monotherapy over placebo in terms of increasing the tolerance threshold to peanut and other food allergen sources (egg, cashew, wheat, milk, walnut, and hazelnut) [65]. 

In addition to monotherapy, anti-IgE is used as an adjuvant therapy for OIT in food allergy in order to reduce the rate of side effects or to enable a faster up-dosing of the food. Here, too, the study results show the efficacy of omalizumab for peanut-allergic children [1, 66] as well as multi-food-allergic patients compared to placebo-treated patients [1, 67]. The data available to date with very good tolerability are promising, but open questions such as the optimal dose as well as the treatment regimen still need to be clarified [1]. While omalizumab is now approved for the indication of food allergy in the USA, this is not the case for Europe/Germany [68]. There is no cure and no immune modulation towards tolerance, so some patients require lifelong treatment [68]. 

The next-generation anti-IgE, ligelizumab has shown very good results in this indication. Due to its biological properties, it will be an interesting new molecule for the treatment of food allergy in the future [1]. Unfortunately, the development program for ligelizumab in food allergy has currently been stopped. 

Dupilumab also has potential for clinical use in the treatment of food allergy due to its ability to downregulate the IgE response during treatment [1, 69]. Initial studies have been started as monotherapy or in combination with OIT. 

In summary, there is still no approved biologic for the treatment of IgE-mediated food allergy in Germany. Although omalizumab has shown efficacy in clinical trials and is currently approved in the USA on the basis of a fast-track procedure for the treatment of food allergy, its use in everyday practice in Germany is still limited to off-label use. 

## Eosinophilic esophagitis 

Another T2-dependent disease is eosinophilic esophagitis (EoE), which, in addition to dysphagia and reflux, can lead to long-term strictures of the esophagus with irreversible damage [70]. Various biologics have so far been investigated in clinical trials for the treatment of EoE. In addition to dupilumab and cendakimab (anti-IL-13), these include well-known biologics such as mepolizumab, which targets IL-5, and benralizumab. Other newer approaches are lirentelimab (anti-Siglec-8) and CALY-002 (anti-IL-15) [71]. 

In Germany, only dupilumab has so far been approved for the treatment of EoE in 2023. However, as dupilumab has not been certified by the Joint Federal Committee as having any additional benefit in EoE, the use of dupilumab in this indication should be determined very carefully and after standard therapy has been carried out. 

## Biologics for the treatment of urticaria 

Omalizumab is at present the only biologic approved for the treatment of urticaria, with several others currently undergoing clinical trials. 

Urticaria is defined as a disease in which wheals, angioedema or both occur suddenly. Chronic urticaria is defined by a disease course of more than 6 weeks. A distinction is made between chronic spontaneous urticaria (CSU) and chronic inducible urticaria (CINDU). The latter has numerous subtypes, some of which are triggered by physical stimuli, such as cold urticaria, and some of which are triggered by other exogenous factors, such as cholinergic urticaria [1]. Treatment is carried out according to the current international guideline for all chronic forms of urticaria using the same algorithm [1, 72]. 

Accordingly, treatment with a non-sedating antihistamine is planned in the first stage at a single dose, and in the second stage up to 4 times the dose if there is no response. In the event of further non-response, omalizumab is also recommended in the third stage and – if the treatment is unsuccessful – cyclosporin A in the fourth stage [1]. The Urticaria Acitivity Score (UAS) was generated to document the clinical response to treatment and was validated for CSU. The UAS records the severity of itching and the number of wheals on a scale of 0 – 3, with a daily maximum value of 6 [1]. The UAS7 is recorded for 7 days to capture fluctuating urticaria, with a maximum score of 42 [1]. 

Although the treatment goal according to the guideline is freedom from symptoms, a weekly UAS7 score of less than or equal to 6 is currently considered a sufficient response. The administration of omalizumab is intended as a third step as an additional therapy to high-dose antihistamines [1]. Omalizumab is a humanized monoclonal antibody directed against IgE with a good safety profile. Its efficacy in CSU has been demonstrated in numerous and comprehensive studies and is 52 – 90% in antihistamine-refractory patients [1, 73, 74, 75, 76], with an adverse event rate comparable to placebo. Nasopharyngitis, sinusitis, colds without probable relation to the medication [1, 75, 76, 77, 78] were the most frequently reported side effects. In contrast to urticaria patients, anaphylactic reactions were reported in asthma patients [1]. 

The drug is now approved as a subcutaneous pre-filled syringe and as a pen for home treatment. A major advantage of the safety profile of omalizumab is that it does not require any preliminary examinations, such as the exclusion of tuberculosis as in the case of TNF-α antagonists. This allows the drug to be used flexibly and safely [1]. The approval stipulates a fixed dose of 300 mg SC, which corresponds to 2 injections of 150 mg, to be administered every 4 weeks [1]. Recent real-life investigation results show that, under certain circumstances, it may be useful to either shorten the interval or increase the dose if there is no treatment response [1, 79, 80]. Patients who are overweight may in particular benefit from an upward dose adjustment [1]. To date, no anti-drug antibodies with effect-limiting properties have been described under omalizumab therapy, which also allows those patients who respond very well to treatment to discontinue the medication after a period of 3 – 6 months on a trial basis without the risk of achieving lower efficacy when restarting [1]. 

Although it is not yet noted in the algorithm in the current guidelines, there are now well-established scientific results that patients without a response to omalizumab 300 mg at 4-week intervals can benefit from a dose increase to 450 mg initially and possibly also to 600 mg [1]. A basic distinction is made between a rapid and a slow response in different patients: In some cases, the response is almost complete as early as 24 hours after the first dose. Other patients show only a slow improvement in UAS7 over the first 3 months of omalizumab therapy [1]. Unfortunately, there is no reliable biomarker as yet which allows for the evaluation of the intensity of treatment response in the individual patient. So far, it is generally true that patients with very low total serum IgE respond less well or not at all. However, even for those patients a time-limited treatment is sensible. For those patients who do not respond to omalizumab, the algorithm of the international guideline provides for the administration of cyclosporin A [1, 72]. In practice, however, cyclosporin A can also be combined with omalizumab [1]. 

Omalizumab has revolutionized the treatment of CSU, but it is also effective in CINDU [1, 77, 81]. Study results – or at least case series – are now available for most of the inducible forms of urticaria [1]. 

Due to the efficacy of omalizumab, the importance of IgE antibodies potentially directed against endogenous structures has further increased. Not only is total serum IgE elevated on average in urticaria patients, but anti-dsDNA, anti-thyroglobulin, and anti-thyroid peroxidase IgE are also found in a number of patients [1, 82, 83]. Against this background, further biologics have been developed that are currently in various phases of clinical testing. A detailed summary of the drugs currently undergoing clinical trials can be found in a current overview [84]. 

The various active substances pursue different pathophysiological approaches. In further studies, it is important to identify biomarkers that identify the subgroups of patients that will respond best to individual therapies. 

The active substances for which approval is expected in the near future are presented here. The phase III trials for dupilumab and remibrutinib are at the most advanced stage. The development of ligelizumab, a humanized IgG1 antibody directed against the Cε3 domain of IgE, has been stopped for the indication urticaria. Compared to omalizumab, it shows a significantly higher inhibition of IgE binding to the high-affinity IgE receptor, but a lower inhibition of IgE binding to the low-affinity receptor CD23 [1, 85]. In phase III, it did not show a significantly better effect than omalizumab. 

Dupilumab has already been discussed several times in this publication and is significant in various diseases from the T2 inflammation group. It has already been approved for various indications. Currently, a phase IIIA study has been successfully completed in comparison with antihistamines in patients aged 6 years and older who were refractory to antihistamines alone. A phase IIIB study in patients of the age of 12 and older who could not be adequately treated with omalizumab under guideline-based therapy was discontinued after an interim analysis. 

Several studies for the use of dupilumab in CSU were positive. The LIBERTY-CSU CUPID study (NCT04180488), a 24-week, randomized, placebo-controlled multicenter phase III study, investigated antihistamine-resistant CSU patients who had either not been treated with omalizumab (CUPID study A) or who had not tolerated omalizumab or had tolerated it only incompletely (CUPID study B) [86]. In CUPID A, dupilumab significantly improved CSU symptoms (UAS7, itch severity score (ISS)7 and hive severity score (HSS)7) at week 24 compared to the placebo group. However, in CUPID B, dupilumab did not reach statistical significance for the primary endpoints in a predefined interim analysis in patients who were refractory to omalizumab. Overall, the safety profile of dupilumab was consistent with what is already known, with few adverse events leading to treatment discontinuation in the dupilumab group compared to placebo. Limitations of the phase III studies included a lack of assessment of durability of response beyond week 24 and a selection bias due to the inclusion of limited numbers of pediatric, adolescent (≥ 6 years), and Black or African-American patients. 

Since early 2024, dupilumab has been approved in Japan for the treatment of CSU in patients aged 12 years and older. Approval for Europe is currently expected in 2024. 

Further innovations in urticaria therapy have been comparatively advanced in the area of small molecules: new selective, reversible, oral bruton kinase (BTK) inhibitors such as fenebrutinib (non-covalent), remibrutinib, rilzabrutinib, and tirabrutinib (all covalent) are currently being tested in trials [87]. Of these, only remibrutinib has so far shown a successful study program for CSU in phase III [88, 89]. Remibrutinib appears to be effective in both antibody-dependent and antibody-independent CSU mechanisms, similar to the efficacy of fenebrutinib in both autoimmune and non-autoimmune CSU types [90]. 

## Biologics for the treatment of hereditary angioedema 

Hereditary angioedema (HAE) is characterized by attacks of recurrent edema of the skin and mucous membranes. Its prevalence is ~ 1 : 50,000 [1, 91]. The autosomal dominant inherited HAE type 1 and type 2 is caused by a genetic defect in chromosome 11, which leads to a deficiency or malfunction of the C1 esterase inhibitor (C1-INH). Other C1-INH-independent types can be attributed to mutations of factor XII, plasminogen or angiopoetin-1, kininogen, myoferlin, and heparan sulphate glucosamine 3-o-sulphotransferase 6. In addition, further mutations that have not yet been identified appear to exist [1, 91]. In addition to C1-INH, the kallikrein-kinin system and bradykinin are pathophysiologically important [1]. Apart from C1-INH preparations, drugs that act on the bradykinin system are therefore also used to treat HAE [1, 91]. Depending on the frequency and severity of the attacks, a distinction must be made in the treatment of HAE patients between acute treatment, short-term and long-term prophylaxis [1, 91]. 

Several C1 esterase inhibitor preparations are approved for both acute treatment and long-term prophylaxis of HAE in the form of intravenous or subcutaneous administration [91]. Icatibant, a selective competitive antagonist of the bradykinin receptor type 2 (B2), can also be administered subcutaneously for acute treatment. It is a synthetic decapeptide with a similar structure to bradykinin [92]. 

Beroltralstat is an oral inhibitor of plasma kallikrein that has been approved for the long-term prophylaxis of HAE from the age of 12 onwards in Germany since 2021. Avoralstat, another oral kallikrein inhibitor, only induced a reduction in the duration of seizures but no significant prevention of HAE attacks [93]. Ecallantide also inhibits plasma kallikrein and is approved in the USA for the treatment of acute HAE attacks in patients aged 12 years and older [94]. 

Lanadelumab is a recombinant, fully humanized monoclonal immunoglobulin G1 kappa light chain antibody that has been available on the German market since 2019 [95]. It is administered subcutaneously every 14 days, although it is possible to extend the dose intervals [1]. The effect is achieved through the highly potent and specific inhibition of plasma kallikrein and thus to a sustained inhibition of plasma kallikrein activity [1, 95]. Since our review was first published [1], several studies have been conducted on the efficacy and safety of lanadelumab for the long-term prophylaxis of HAE attacks in patients aged 12 years and older with confirmed C1 esterase inhibitor-induced HAE. The actively treated groups showed a significant reduction in the attack rate and an increase in the percentage of patients without attacks compared to the placebo group. Treatment with lanadelumab was generally safe and well tolerated. The most common treatment-related adverse events were local reactions at the injection site [1, 95, 96, 97]. Good efficacy and tolerability have now also been demonstrated in children aged 2 – 11 years [98]. 

Garadacimab is a human IgG4 antibody for subcutaneous administration that inhibits activated factor XIIa and thus also bradykinin formation by binding to it. In a 6-month phase III study involving 65 patients with C1-INH-dependent HAE, a significant reduction in the number and severity of attacks was achieved in the actively treated group (n = 39) compared to the placebo (n = 26). The dose was 400 mg once, followed by 5 monthly 200-mg doses. The most common adverse effects were respiratory tract infections, nasopharyngitis, and headache. No increased risk of bleeding or thromboembolic events was observed [99]. Marketing authorization in Germany is expected at the beginning of 2025. 

## Hypersensitivity reactions to biologics 

The classification of adverse reactions to biologics by Werner Pichler (2006) into five categories (alpha to epsilon) is still valid and practicable today [1, 100]. 

The most common adverse reactions to biologics are those of the alpha type and are attributable to the direct pharmacological effect, the immune stimulation through cytokine release (“on-target”). They are substance- and dose-dependent, occur on initial application and decrease in severity as treatment continues. They are called “infusion reactions” and include the “cytokine release syndrome” [1]. 

Hypersensitivity reactions, including type I to IV allergies, i.e., immune reactions to the therapeutic protein, represent type beta. They are so-called “off-target” reactions, unpredictable, not dependent on the administered dose, and, with the exception of anaphylaxis to cetuximab, do not occur on initial application [1]. 

Type gamma includes those side effects in which immune deviation occurs as a result of the biologic. These include the development of opportunistic infections (e.g., activation of tuberculosis by TNF-α blockers), the development of a neoplasia, the de novo induction of autoimmune diseases such as systemic lupus erythematosus, the exacerbation of psoriasis or the development of (atopic dermatitis-like) eczema under biologic therapy of psoriasis [101] or bullous pemphigoid [102]. Dupilumab therapy of patients with atopic dermatitis, for example, can lead to psoriasis or psoriasiform eczema [103]. 

Paradoxical reactions have been described after biologic therapy of dermatological diseases. They are defined as the development of a new or the exacerbation of a pre-existing immune-mediated disease after the start of biologic therapy. This may include exacerbation of the treated disease [104]. Such paradoxical reactions often manifests on the skin (psoriasis, psoriasiform eczema, eczematous reactions, alopecia areata, sarcoidosis-like or granulomatous skin changes, rosacea, lupus-like reactions). They are therefore most likely to be classified as type gamma. They mainly occur after treatment with biologics that address cytokine signaling pathways, i.e., inhibitors of TNF-α and cytokines such as IL-12/23 (p40), IL-17A (and IL-17R), IL-23 (p19), and IL-4Rα. 

The delta type describes reactions that are triggered by structural similarity of binding or antigen molecules, such as acneiform exanthema under cetuximab therapy. 

Type epsilon stands for the non-immunological side effects. Dry eyes and conjunctivitis, which can be caused by dupilumab therapy for atopic eczema, are representatives of this category [105]. 

Reactions of type alpha and beta can be life-threatening and are accompanied by symptoms that are similar to those of anaphylaxis by definition. To date, the non-IgE-mediated reaction and the cytokine storm following biologic therapy are not fully understood pathophysiologically/mechanistically, which makes classification difficult [1, 106]. Due to the fact that they can be life-threatening, the triggering biologics are usually discontinued without further diagnosis if symptoms develop, a decision that can, however, pose a problem for patients with regard to the underlying disease [1]. Switching to another biologic with a similar indication and efficacy profile is not always immunologically unproblematic and, therefore, may not be safe. 

We therefore need in vitro test methods to detect the presence and effect of neutralizing anti-drug antibodies (ADA) including IgE antibodies and, if necessary, to identify cross-sensitivities, thereby ensuring better therapy management. For example, the treatment of cytokine release syndrome is different from that of anaphylaxis. In the case of anaphylaxis, premedication with antihistamines and glucocorticoids will not help, and the risk of subsequent anaphylaxis at the next application is high [1, 106]. 

A comprehensive literature research a few years ago revealed that the classification of hypersensitivity reactions to biologics is not harmonized throughout the various databases, partly pharmacovigilance surveys, which makes it difficult to determine the prevalence of “true” allergic and anaphylactic reactions to biologics [1, 107, 108]. In addition, the symptomatology of anaphylaxis to different biologics can vary [1, 106]. Only the careful characterization of patients with such reactions and the documentation in registries will be able to provide a solution to this problem [1]. 

One of the best-known examples of the allergenicity of biologics is cetuximab, on which a new epitope, galactose-α 1,3-galactose (α-Gal), a disaccharide, was first identified and also identified as the cause of delayed anaphylaxis to mammalian meat and meat-products after anaphylaxis occurred following initial application of this oncological biologic therapy [1, 109]. Cetuximab is one of several chimeric therapeutic antibodies currently in use (consisting of both a murine and human part, of which the murine part holds the α-Gal epitope in the case of cetuximab) [1]. Other chimeras are rituximab and infliximab. A higher degree of humanization of the biologics was, therefore, aimed at in order to reduce their immunogenicity/allergenicity. Only for infliximab has a further association with anti-α-Gal-IgE been described to date [1, 110]. Anti-α-Gal IgE can now be routinely determined in patients’ serum using the ImmunoCAP method (Phadia AB, Thermo Fisher Scientific, Uppsala, Schweden) with bovine thyroglobulin as allergen, but experimental multiplex methods based on different α-Gal-carrying proteins have been developed since then [111]. 

Tick bites are presently considered the most important route of sensitization [112]. For patients living in endemic areas with a high prevalence of α-Gal sensitization, it makes sense to determine the IgE antibodies against α-Gal before administering cetuximab [1, 109, 113]. Other international research groups have also experimentally demonstrated IgE against various biologics [106] and shown their clinical relevance: patients with IgE against an administered biologic in serum and/or in the skin test reacted more rapidly (3^rd^ dose) and more strongly when re-exposed to this biologic [1, 114, 115]. To date, no routine procedure is available for the determination of anti-drug IgE to all biologics [1]. 

The degree of humanization of biologics reduces their immunogenicity. However, even the use of fully human sequences can trigger immune responses [100, 116]. IgG-type ADAs can neutralize treatment efficacy. IgG diagnostic tests have been available for this indication for some time. 

Anti-infliximab IgG, IgE, and IgM are detectable in sera from patients with infliximab anaphylaxis during infusions [1, 114, 117], the clinical relevance of IgM remaining unclear. Anti-infliximab antibody detection can be valuable for risk assessment of the development of a hypersensitivity reaction [1, 115, 116, 118]. 

**Figure 1:**
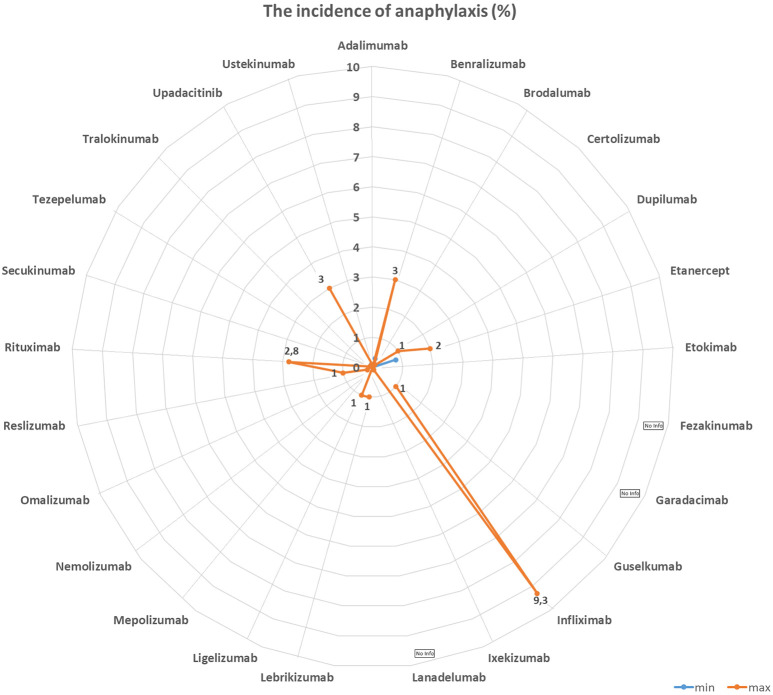
The incidence of anaphylaxis (%).

**Figure 2:**
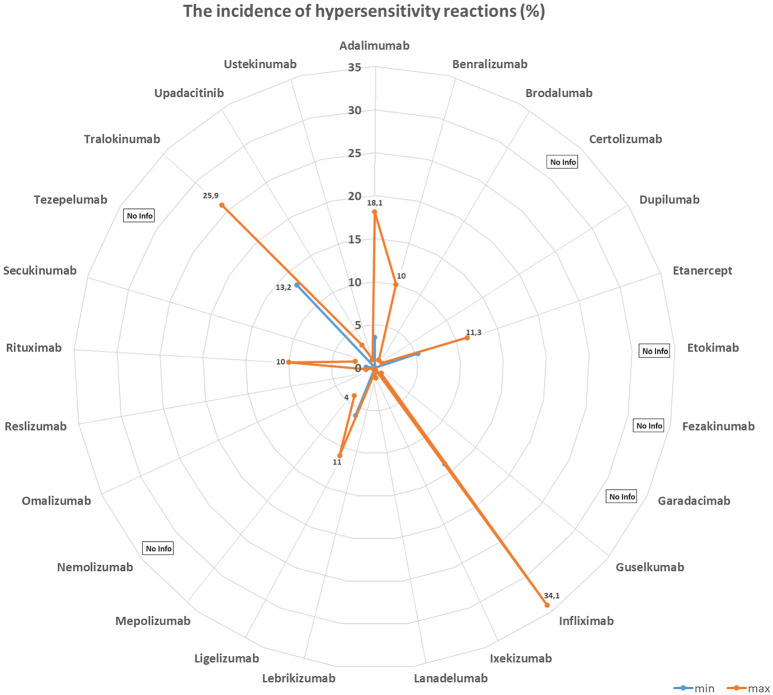
The incidence of hypersensitivity reactions (%).

**Figure 3:**
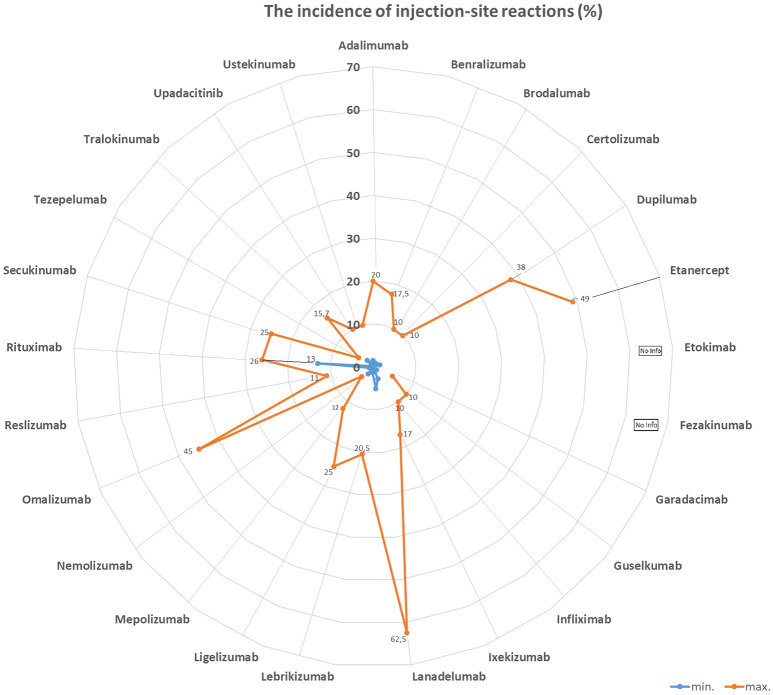
The incidence of injection-site reactions (%).

**Figure 4:**
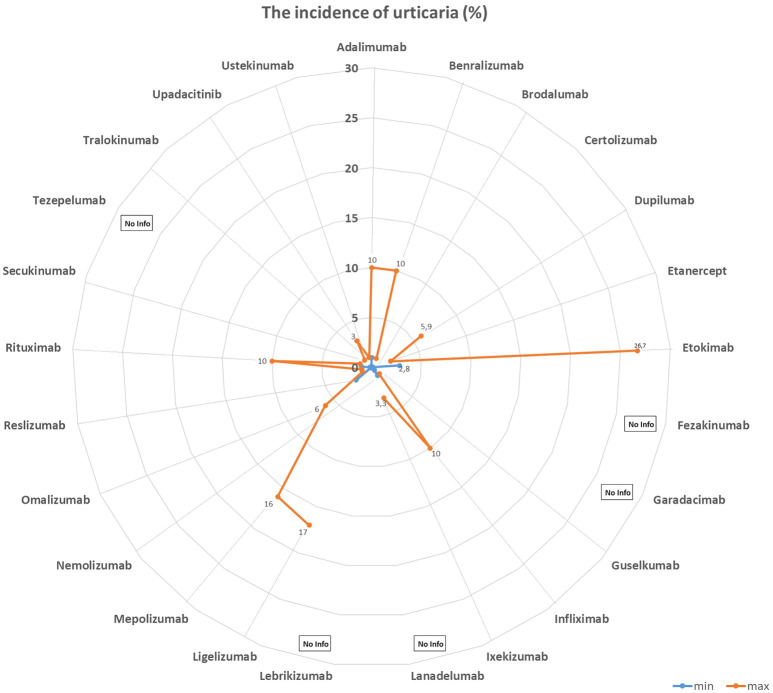
The incidence of urticaria (%).

Table 1 and Figures 1, 2, 3, and 4 summarize the frequencies and phenotypes of hypersensitivity reactions to the biologics for the treatment of atopic diseases listed in this review article. Serum sickness-like reactions have been described for dupilumab [1, 119] and rituximab [120]. 

For the diagnosis of the different phenotypes of hypersensitivity reactions, a precise medical history is crucial. This should include the following: Type of the reaction, time of occurrence, and progression of the reaction during the course of therapy, dependence or independence from the administered dose, method of application, duration of therapy and therapy breaks, information on possibly living in an α-Gal-sensitization endemic area, and mammalian meat allergy [1]. 

In addition, skin testing (prick and/or intradermal test) with the suspected biologic provides information about the presence of IgE sensitization in the skin. However, skin tests with the drug represent off-label use, about which the patient should be informed and give written consent. In general, allergy diagnostics should be performed within a period of 4 – 6 weeks after the event in order to be informative [1, 108]. A few clinical research groups are working on the establishment of assays for IgE against biologics. According to own studies, the detection of so-called pre-existing IgE antibodies before the start of treatment with biologics is particularly important for the decision in favor of safe treatment, but tests to monitor IgE (ADA) development in the course of treatment are also important for managing the risk of developing anaphylaxis [1, 121, 122]. Anti-biologic IgE tests are also suitable for detecting possible cross-reactions via antigen or epitope similarities between biologics, which is of decisive importance for the choice of an alternative therapeutic agent in the event of a hypersensitivity reaction [108]. There is an increasing number of publications on cell-based tests such as the basophil activation test with biologicals [123, 124], which already appear to be able to determine the suitability/safety of the potential alternative biologic with sufficient certainty. 

Apart from side effects mediated by the active substance, additives such as polysorbate, mannitol, albumin, latex, trometamol, and papain [108, 125] should also be included in the allergy tests [1]. 

For the management of biologic hypersensitivity reactions, it is important to realize that re-exposure after a pause in therapy led to sensitization, at least for infliximab treatment [115]. In order to prevent reactions, it is important to identify possible risk factors. Atopy status or the presence of drug reactions in the medical history do not appear to be clinical risk factors for hypersensitivity reactions to biologics. Smoking and infections during therapy as well as HLA-DQA1*05, however, appear to be associated with the formation of ADA. The underlying disease may be equally important, especially if it is associated with a highly activated B-cell status and strong expression of costimulatory molecules on dendritic cells in patients with immune-mediated diseases [116]. 

## Biologics therapy and vaccinations 

The use of biologics strongly interferes with immune regulation, not always only in the sense of the intended indication, so that the question arises as to whether this has an effect on the immune response to infectious agents, i.e., bacteria, viruses, fungi, and parasites, i.e., whether there is an increased susceptibility to infection under the respective biologics therapy. In general, vaccination programs against many pathogens are carried out very successfully today to prevent (these) infections [1]. 

The biologic therapies approved by the authorities intervene in immune cascades in a targeted manner. A distinction can be made between the elimination of circulating B cells (anti-CD20 therapy), the neutralization of important acute inflammatory messengers such as TNF-α or IL-1, as well as the targeted influencing of certain (specialized) T-cell functions (e.g., IL-5, IL-4/IL-13, IL-23, IL-17, IL-12/IL-23). 

Vaccinations are an effective instrument of preventing new infections or the reactivation of “old” silent or latent infections. When an immune system is specifically modulated by biologics, the question arises as to whether the vaccination can have the desired effect. On the one hand, the vaccination effect relates to the formation of a corresponding antibody reaction (seroconversion) or the development of a specific T-cell response. In the case of live attenuated vaccines, there is also the question of whether the vaccine actually leads to an infection in the modulated host immune system. Most data is available on anti-TNF-α therapy for rheumatoid arthritis, and with regard to the type of vaccination, it is the flu vaccine (influenza vaccine). Four studies were able to show that the antibody response to influenza vaccination is attenuated under anti-TNF-α therapy [126, 127, 128, 129], whereas three studies were able to demonstrate a normal vaccination response [130, 131]. Further studies show that human papilloma virus (HPV) vaccination only leads to an attenuated T-cell-dependent immune response [132], whereas the antibody response to pneumococcal vaccination was normal [133, 134]. 

For this reason, the immune status with regard to these pathogens (and other infections) should be investigated before treatment with TNF antagonists. There is a pragmatic suggestion for the respective diagnostic tests in the literature [135] (Table 2), and this can also be applied before treatment with biologics for allergy and asthma [1]. It is advised to check the immune status with regard to these important viruses (and bacteria) before starting a biologics therapy and, if necessary, to refresh vaccinations or catch up on missing vaccinations before initiating the respective biologics therapy [136]. 

The European Association of Rheumatologists (EULAR) developed recommendations for the vaccination of patients with rheumatic diseases undergoing biologics therapy in 2011 [1, 137]. These were updated in 2015 [138]. In addition to statements for the pediatric population, a further recommendation for adults was published in 2020 [139]. The basic recommendations listed there are essentially also shared by experts in the field of dermatology and allergology [139]. These recommendations apply to the following biologics: anti-TNF-α; anti-IL-12/IL-23; anti-IL-23; anti-IL-17; anti-IL- 4Rα. A basic distinction is made between (protein) inactivated vaccines and live-attenuated vaccines. The former category includes the pneumococcal vaccine, inactivated influenza vaccines, vaccines against *Haemophilus influenza* type B, hepatitis A and B, HPV, as well as tetanus and diphtheria toxin, and the acellular pertussis vaccine. 

Vaccinations should be administered as clinically indicated or recommended. The situation is different for the live-attenuated vaccines. These include measles, mumps, rubella, oral poliomyelitis, oral typhoid fever, yellow fever, and varicella zoster vaccines. These should explicitly not be administered during biologics therapy. 

One important vaccination is the tetanus vaccination. In this regard, it was shown for the application of dupilumab and the two anti-IL-13 antibodies tralokinumab and lebrikizumab that the development of the anti-tetanus antibody vaccination responses as well as other immune responses are not impaired [140, 141, 142]. It can therefore be concluded that patients receiving dupilumab can be vaccinated with inactivated or killed vaccines at the same time [1]. Among the vaccinations against viral infections, that against influenza is of particular importance, especially as patients with asthma have an increased risk of (severe) influenza infection. Treatment with monoclonal anti-IL-5 receptor antibodies (benralizumab) has been shown not to impair the vaccine-induced antibody response to seasonal influenza vaccination in adolescents and young adults with moderate to severe asthma [1, 143]. 

In recent years, the focus has been on overcoming the corona virus pandemic, which was induced by the SARS-CoV-2 virus and led to COVID-19 disease. In the phase III trials that were conducted with various COVID-19 vaccines and ultimately led to the marketing authorization of various protein- and mRNA-based vaccines, patients on immunosuppressant therapy or patients with significant autoimmune disorders were excluded. It was therefore necessary to conduct further studies to investigate the extent to which patients with underlying immunological diseases and/or biologic therapies interact with the available vaccines. From this, the following basic international recommendation was derived, which also applies to patients with allergies and asthma: First of all, it must be noted that the vaccination recommendation applies to all patients even with these underlying diseases or previous therapies. Data obtained from the – admittedly – relatively small number of cases suggests that the vaccination can also be carried out in this patient group without any specific additional risk. This also applies to the question of the efficacy of the vaccination in this group; there are only a few data on this, but they have not yet shown any significant loss of efficacy [144] with the exception of a small recently published study describing a lower immune response to mRNA vaccines when treated with mepolizumab, benralizumab, or dupilumab [145]. 

In conclusion, it is generally not possible to generalize results from one vaccine to another, as vaccinations against different pathogen classes (viruses and bacteria) by very differently configured vaccines (e.g., live attenuated and dead vaccines, significance of the added adjuvant) also trigger different immunological strategies. Therefore, there is still a considerable need for further clinical studies [1]. 

## Biologic therapy in patients with uncertain SARS-CoV-2 infection status 

In the context of infectious pandemics such as the COVID-19 pandemic, concerns may arise regarding the application of immunosuppressive drugs and/or immunomodulating biologics. These mainly concern the theoretical consideration of whether viral infections may contribute to more severe courses or whether there are possible immunological interactions and potential risks of these biologics if the immune response is influenced by the biologic [146, 147, 148]. In allergology, patients mainly receive biologics that inhibit type 2 immune responses via different mechanisms [146, 147, 148]. There is uncertainty about the administration of biologics in the case of an infection with, e.g., SARS-CoV-2 and also about the treatment recommendations in the context of vaccinations (see above). Position papers from the German [148] and European [146] societies of allergology have summarized the possible mechanisms and current knowledge in this regard. 

As a recommendation, the use of biologics for the treatment of bronchial asthma, atopic dermatitis, chronic rhinosinusitis with nasal polyps, EoE, hypereosinophilia syndrome, and spontaneous urticaria in patients without suspected or confirmed SARS-CoV-2 infection should be continued as usual. Treatment should aim to control difficult-to-treat allergic and atopic conditions as much as possible with an appropriate therapy and to avoid the need for systemic glucocorticosteroids. If SARS-CoV-2 infection is confirmed or reasonably suspected, the treatment decision towards a biologic should be determined by individually weighing up the benefits and risks for the individual patient, with the patient being included in the decision-making process [146, 147, 148]. The relevant literature also states that:

- Numerous studies have shown that there is no increased risk of COVID-19 infection or poorer treatment outcome in patients treated with T2-addressing biologics.
- For dupilumab, it has even been described that therapy with the antibody leads less frequently to severe or fatal courses than in patients in comparison groups [149, 150, 151, 152].
- No serious reactions in terms of treatment safety have been reported in patients treated with T2-addressing biologics under SARS-CoV-2 vaccination.
- Allergy-like symptoms in the skin and respiratory tract after SARS-CoV-2 vaccination have been described but should not discourage vaccination as they are apparently rare and a causal relationship has not been established, as this is based on sporadic spontaneous case reports while billions of doses of COVID-19 vaccines have been administered worldwide.
- The use of T2-addressing biologics had no effect on the antibody response after SARS-CoV-2 vaccination.

## Biologics during pregnancy and in childhood 

Most scientific publications and studies on the subject of biologics in pregnancy can be found on autoimmune or inflammatory chronic diseases, such as rheumatoid arthritis, lupus erythematosus, or psoriasis vulgaris. Active autoimmune diseases as such carry an increased risk of adverse maternal and fetal events, such as pre-eclampsia, miscarriage, intrauterine growth retardation, premature birth, or low birth weight [1, 153]. Inadequately controlled bronchial asthma also increases the risk of complications for mother and child. Sufficient control of disease activity is, therefore, crucial. For example, the treatment goal for rheumatoid arthritis is to have no to low disease activity before conception, as the negative effects of systemic glucocorticosteroids and non-steroidal anti-inflammatory drugs in particular must be considered [1, 154]. 

Results from case reports and registry data evaluations of treatment with TNF antagonists, which have been approved for many years for the therapy of rheumatological diseases as well as psoriasis vulgaris, have so far shown no evidence of an increased number of spontaneous abortions or malformations [1, 155]. As a result, the use of TNF inhibitors such as infliximab, adalimumab, and etanercept is recommended in pregnancy up to week 20 [1]. The Fab fragment of the TNF antibody certolizumab, which cannot cross the placental barrier, was classified as safe for the entire pregnancy [1, 156, 157, 158]. For ustekinumab and vedolizumab, initial data from a large cohort of pregnant inflammatory bowel disease (IBD) patients show good safety data for mother and child [159]. With regard to other biologics, such as secukinumab, ixekizumab, and brodalumab, only limited data are available to date, meaning that use during pregnancy is currently not recommended, primarily for precautionary reasons [1, 158]. 

The biologic omalizumab, which has been authorized and used for the longest time in allergology, was investigated in the so-called Expect study [1, 160]. Here, 250 women with asthma who received omalizumab during pregnancy were examined. The data show no evidence of an increased risk of congenital malformations. Nevertheless, there are still no corresponding recommendations in the international guidelines [1]. However, the Institute of Clinical Pharmacology and Toxicology (Charite Berlin) states on its website embryotox: “Omalizumab can be used in pregnancy if other safer alternatives fail, as effective disease control is an important prerequisite for an undisturbed pregnancy. However, this requires a careful individual risk-benefit assessment” (https://www.embryotox.de/ download August 22, 2024). 

For dupilumab, there is comparatively less published data on application during pregnancy [161, 162, 163]. In 29 patients who received dupilumab for a median of 6 weeks in the first half of (mostly unplanned) pregnancy, there were no complications [161]. Furthermore, 7 case histories were published (summarized in [163]. Here, 1 premature birth and 1 child with low birth weight were reported, but otherwise no complications [163]. According to these first published data, the application of dupilumab does not appear to be very critical in the early stages of pregnancy. This is of particular clinical relevance for unplanned pregnancies under dupilumab therapy. 

Overall, the natural Th2 polarization of the immune system in advanced pregnancy should contribute to the normal course of pregnancy by creating a tolerogenic environment for the fetus. In this respect, it is important to generate sufficient evidence on the frequency of miscarriages and premature births under dupilumab therapy in order to be able to make a reliable risk assessment. The deliberate use of dupilumab during pregnancy is currently not recommended in position papers and guidelines on atopic dermatitis [164, 165]. There is also no formal authorization for application during pregnancy. Two prospective observational studies on this topic are currently underway and should be completed in 2026 and 2027 (https://clinicaltrials.gov/study/NCT04173442; https://clinicaltrials.gov/study/NCT03936335). 

The other antibodies used in allergology, such as benralizumab and reslizumab, are not yet recommended for use during pregnancy due to a lack of data [1]. Particularly in the third trimester, transfer of antibodies to the child via the placenta is possible. On the other hand, unstable chronic diseases due to the increased use of oral corticosteroids, for example, must be taken into account [1]. In the future, further register-based data or case-control studies are required in order to establish the evidence for the safe use of biologics in pregnancy, also in the field of allergy [1]. 

## Biologics: Therapy for children 

The use of biologics in children is individual and dependent on the indication: For example, omalizumab can be used in children with allergic asthma from the age of 6 according to the Summary of Product Characteristics, but in CSU only from the age of 12. More biologics have been approved for children: Dupilumab has been approved since 2023 for the treatment of atopic dermatitis in children from 6 months of age and for the treatment of allergic bronchial asthma from 6 years of age [166]. Mepolizumab is also approved from 6 years of age onwards for children with severe, refractory asthma. Lanadelumab is approved for children aged 12 years and older; good efficacy and tolerability were also recently demonstrated for the 2 – 11 years age group for the indication of HAE [98]. 

## Future biologics for atopic diseases 

As the range of applications and the number of preparations increase, the authors would like to take a look at what other targets could play a role in the future. 

### Bronchial asthma 

In bronchial asthma, established paths are explored further. IL-4 is already known as a target for dupilumab. As a new IL-4R antagonist, CM-310 is currently undergoing a phase II trial [167]. The inhibition of IL-5 in severe eosinophilic bronchial asthma is also being investigated. Here, substance 610 is currently investigated in phase II [168] and depemokimab in phase III trials [169, 170, 171]. 

FB825, an antibody against membrane-bound IgE on B cells, is currently showing an alternative mechanism in phase II trials [172] of how IgE can be used as a target marker to induce apoptosis. 

New pathways are opening up in the area of type 2 low-bronchial asthma, a variant in which typical markers of type 2 inflammation are absent. IL-6 is currently a focus of studies. FB704A as an IL-6 inhibitor specifically for severe neutrophilic bronchial asthma is now investigated in phase II trials [173] after a positive phase I [174], as is clazakizumab [175]. 

Itepekimab, an IL-33 inhibitor, acts much earlier in the inflammatory cascade. In phase II studies, it showed similar effects in lowering the percentage of patients with a loss of asthma control as dupilumab [176]. In addition, the evaluation of the now completed phase II [177] study of tozorakimab with the same target is awaited. 

Progress in investigating the effects of tyrosine kinase inhibitors (TKIs) on atopic diseases is not only being made in the field of atopic dermatitis. Masitinib as a c-kit pathway TKI showed a significant reduction of 50 – 70% in the asthma exacerbation rate in phase III trials [178, 179]. Rilzabrutinib as a Bruton-TKI is currently still undergoing phase II trials [180]. 

Phase II trials for the CD4-IgG1 antagonist tregalizumab, originally planned for the indication rheumatoid arthritis, were discontinued due to a lack of efficacy in bronchial asthma [181]. 

The respective manufacturer has also discontinued the further development of the IL-1RL1 antagonist melrilimab in the phase IIa trial [182]. 

Another candidate for future asthma therapeutics is amlitelimab, which has already shown promising data as an OX40 ligand inhibitor in the indication of atopic dermatitis and is now in a phase II trial for bronchial asthma [183]. Povorcitinib as a JAK1 inhibitor is also attempting to transfer the success of JAK inhibitors from atopic dermatitis to bronchial asthma in a phase II trial [184]. 

### Atopic dermatitis 

Further promising therapy options are also currently being investigated for treatment of atopic dermatitis. 

In phase III studies, treatment with nemolizumab as an IL-31R antagonist was characterized above all by very rapid relief of itching, while the improvement in skin symptoms was delayed [185]. The drug was already approved in Japan in 2022 for patients aged 13 years and older with refractory itching in atopic dermatitis [186]. 

Another promising target could be the inhibition of the OX40/OX40 ligand axis, which plays an important role in the interaction between dendritic cells and T cells. 

With amlitelimab as an OX40 ligand inhibitor, the phase II study [187] not only showed improvements in the validated Investigator Global Assessment (vIGA) score, but also a continued clinical improvement up to week 24 [188]. Rocatinlimab with the same target molecule achieved a significant improvement in the EASI score after 16 weeks in a phase IIb study at all investigated doses. The achievement of EASI-75 was also maintained in the 20-week follow-up interval [189]. 

For telarzolimab, a phase IIa study showed that 2 intravenous doses administered 4 weeks apart led to a significant reduction in the mRNA expression of cytokines of the Th2, Th1, and Th17/Th22 axis in skin biopsies compared to placebo up to 42 days after the last dose, as well as to a significantly higher achievement of EASI-75 [190]. Thus, this substance class could possibly have disease-modifying effects. 

One step higher in the cascade are anti-TSLP antibodies, which should then also exert an inhibitory effect on the OX40/OX40 ligand axis. While a marketing authorization for asthma exists, a pronounced placebo effect was shown in the control group in phase II for atopic dermatitis, which could be a possible explanation for the lack of proof of effect [191, 192]. 

Other substance classes currently being investigated include further IL-4Rα antagonists, IL-13Rα subunit antibodies (eblasakimab), IL-5 antibodies (benralizumab), IL-33 antibodies (tozorakimab), antibodies against membrane-bound IgE on B cells (FB-825), IL-22 antibodies (LEO 138559), anti-IL-18, Siglec-8 agonists (lirentelimab) and Bruton TKIs. 

In phase II studies, secukinumab [193] as an IL-17A inhibitor, risankizumab [194] as an IL-23 subunit p19 inhibitor, etokimab [195] and astegolimab [196] as IL-33 inhibitors, and spesolimab [197] as an IL-36R antagonist did not prevail over placebo. 

While systemic JAK inhibitors have now entered clinical practice, the marketing authorization of topical JAK inhibitors is expected. For various molecules (delgicitinib, tofacitinib, ruxolitinib, cerdulatinib, ifidancitinib), a significant improvement in EASI was shown compared to placebo and in some cases even compared to systemic JAK inhibitors with a favourable side effect profile [198]. 

### Chronic rhinosinusitis 

CM-310 as an IL-4R antagonist showed significant improvements in the nasal polyp score (NPS) [199] and nasal congestion score (NCS) in the phase II study [200] and is currently in a phase III study [201]. The IL-4Rα inhibitor GR1802, which works on the same target molecule, is currently completing a phase II trial [202]. 

Benralizumab as an IL-5R inhibitor was also able to show significant improvements in NPS and nasal blockage score (NBS) compared to placebo in the phase III trial, but not in the sino-nasal outcome test (SNOT-22) and the time to the next functional endoscopic sinus surgery (FESS) and/or systemic corticosteroid administration [203]. The results of the currently ongoing phase III trials [204, 205] of the IL-5R antagonist depemokimab will therefore be all the more exciting. 

Starting high up in the inflammatory cascade, it remains to be seen whether tezepelumab can also demonstrate its success as an anti-TSLP antibody for CRS in the ongoing phase III trial [206]. 

In contrast, the IL-33 inhibitor etokimab did not prevail over placebo in the improvement of SNOT-22 in Phase II [207]. 

### Chronic spontaneous urticaria 

All doses of the Bruton TKI remibrutinib prevailed in the phase II study with regard to the reduction of the urticaria activity score (UAS) compared to placebo [208]. The drug is currently in phase III trials [209, 210, 211, 212, 213, 214]. 

Fenebrutinib from the same drug group showed a dose-dependent improvement in the urticaria activity score over 7d (UAS7) after week 8 in the completed phase II study. Transient transaminase elevations were spontaneously reversible [215]. 

Although ligelizumab as an IgE inhibitor showed a reduction in the hive severity score (HSS) and itch severity score (ISS) comparable to omalizumab in the phase III study, it was not superior to omalizumab, which is why the focus is currently on possible use in the area of food allergies or chronic inducible urticaria [216, 217]. 

The phase II study results of benralizumab as an IL-5R inhibitor in the ARROYO study showed no clinical effect over placebo [218]. The results of tezepelumab as an anti-TSLP-AK in the INCEPTION study [219] are still pending. 

In contrast, although dupilumab achieved a significant reduction in urticaria activity in omalizumab-naïve patients with CSU that could not be controlled by antihistamines, this was not the case in omalizumab non-responders [220]. 

Further candidates for future CSU therapeutics with current study activity are shown in Table 3. 

### Eosinophilic esophagitis 

The results from the phase II/III trial with the Siglec-8 agonist lirentelimab are awaited [226]. In a randomized, double-blind, placebo-controlled, multicenter study, mepolizumab was unable to achieve the primary endpoint of an improvement in dysphagia symptoms when compared to placebo [227]. 

Further candidates for future EoE therapeutics with studies currently ongoing are shown in Table 4. 

### Eosinophilic gastritis 

In the phase III study, the IL-5R inhibitor benralizumab achieved a significant histological remission in mucosa samples versus placebo, reduction of eosinophils both locally and systemically, but no significance in the symptom survey (SODA) and the Gastric Endoscopic Score [232]. 

After completion of the phase III trials [233, 234], the company developing the substance clinically announced that it would withdraw from research into eosinophilic gastritis with the drug lirentelimab as a Siglec-8 agonist and investigate further use in atopic dermatitis and CSU, as the expected endpoints for the symptom scores were not achieved in the original indication [235]. 

Other candidates currently being investigated for this indication are dupilumab and cendakimab (an IL-13 inhibitor) [236, 237]. 

### Food allergies 

Other currently investigated drugs with significantly less evidence in the area of food allergies are dupilumab [238], the anti-IgE antibody ligelizumab (currently in a phase III trial for peanut allergy) [239], agammaglobulinemia TKI acalabrutinib (completed phase II [240]) and etokimab (anti-IL-33 antibody) [241, 242]. 

## Conclusion 

The continuous development of biologics in the targeted therapy of atopic diseases has confirmed pathomechanistic hypotheses on the significance of certain therapeutical target molecules in atopic diseases, urticaria, and angioedema. It is certainly worthwhile continuing to extend indication of existing biologics and to develop new substances in well-designed studies. This could provide important novel insights into the underlying pathophysiology and immunology of atopic diseases, urticaria, and angioedema. 

## Acknowledgment 

This paper is a comprehensive update of the publication of the DGAKI Working Group on Biologics and New Pharmaceuticals entitled “Biologics in atopic diseases: Indication, side effect management and new developments” by Jappe et al. in Allergologie 2021; *41:* 54-80 (as well as in Allergologie select) [1]. Where information was adopted directly, it was provided with the corresponding literature citation. 

## Authors’ contributions 

UJ: designed, coordinated, wrote the manuscript, wrote the chapter hypersensitivity reactions to biologics and in parts food allergy, edited and revised the whole manuscript, edited Table 1. KCB, CT: wrote and revised the chapter bronchial asthma. ST: wrote and revised the chapter atopic dermatitis. TW: wrote and revised the chapter atopic dermatitis, in parts SARS-CoV-2-infection status, pregnancy and childhood, edited and revised the whole manuscript. FB: wrote and revised the chapter pregnancy and childhood. MWo: wrote and revised the chapter food allergy and EoE. MWa: wrote and revised the chapter chronic sinusitis with nasal polyps. TZ: wrote and revised the chapter urticaria, edited the whole manuscript. RT: wrote and revised the chapter hereditary angioedema. HR: wrote and revised the chapter vaccinations, Table 2. LK: wrote and revised the chapter SARS-CoV-2-infection status. SPS, VF, ST: wrote and revised the chapter future biologics, Tables 3 and 4. AG: designed and produced the table 1 and the Figures 1 – 4. 

## Funding 

No extra funding for the creation of this manuscript. 

## Conflict of interest 

CT, AG, VF, KCB, and FB have no conflict of interest. 

UJ received hotel accommodation and meals for a lecture and for leading a workshop organized by ALK Abello. The fee went to her organization, the RCB. In addition, another hotel night and dinner were recently provided by ALK Abello. Her research on molecular allergology is funded by the Federal Ministry of Education and Science, the Federal Ministry of Food and Agriculture (BMEL), the German Research Foundation and the Kanert Foundation: all outside the topic of this article. The Federal Ministry of Technology, Economics and Technology has funded their research on assays for the detection of anti-drug antibodies via the AiF-ZIM program. 

ST reports support for consultancy, lectures, and other scientific activities from AbbVie, Janssen/ JNJ, Leo Pharma, Lilly, Novartis, and Regeneron/Sanofi outside the submitted work, memberships: DGAKI, DDG, EAACI. 

RT received research support from Sanofi-Genzyme and the Hautnetz Leipzig/Westsachsen e.V. as well as fees for lectures and consultations from ALK-Abello, Takeda, Novartis, Sanofi-Genzyme, Abbvie, and support for congress visits from Takeda. 

LK reports grants and/or personal fees from Allergopharma, MEDA / Mylan, HAL Allergie, ALK Abelló, LETI Pharma, Stallergenes, Quintiles, Sanofi, ASIT Biotech, Lofarma, Allergy Therapeut., AstraZeneca, GSK, Inmunotk and Cassela med outside the submitted work; and memberships: AeDA, DGHNO, German Academy of Allergology and Clinical Immunology, HNO-BV, GPA, EAACI. 

MWo reports support for consultancies, lectures and other scientific activities from ALK-Abelló Arzneimittel GmbH, Abbvie, Eli Lilly, Mylan Germany GmbH, Bencard Allergie GmbH, Novartis AG, Biotest AG, Sanofi-Aventis Deutschland GmbH, HAL Allergie GmbH, DBV Technologies S.A, Aimmune Therapeutics UK Limited, Regeneron Pharmaceuticals, Inc, Stallergenes GmbH. 

S. Seurig reports support for consultations, lectures, and other scientific activities by Allergopharma, ALK-Abelló Arzneimittel GmbH, AstraZeneca, Takeda outside the submitted work, memberships: DGAKI, DGP, EAACI 

TZ reports support for consultations, lectures and other scientific activities by AstraZeneca, AbbVie, ALK, Almirall, Astellas, Bayer Health Care, Bencard, Berlin Chemie, FAES, HAL, Henkel, Kryolan, Leti, Lofarma, L’Oreal, Meda, Menarini, Merck, MSD, Novartis, Pfizer, Sanofi, Sanoflore, Stallergenes, Takeda, Teva, UCB as well as responsible participation in the following organizations: Committee member, WHO initiative “Allergic Rhinitis and its Impact on Asthma” (ARIA), Member of the Board, German Society for Allergy and Clinical Immunology (DGAKI), Head, European Centre for Allergy Research Foundation (ECARF), Secretary General, Global Allergy and Asthma European Network (GA^2^LEN), Member, Committee on Allergy Diagnosis and Molecular Allergology, World Allergy Organization (WAO). 

TW reports support for consultancy, lectures, and other scientific activities from AbbVie, ALK Abello, Almirall, Astellas, Bencard, Galderma, Janssen/JNJ, Leo Pharma, Leti, Lilly, Novartis, Pfizer, Regeneron/Sanofi, Stallergen. 

MWa has received fees for consulting, lectures or research support from the following companies in the past 3 years: Allergopharma, ALK-Abelló, AstraZeneca, CSL Behring, Genzyme, GSK, HAL Allergie, Infectopharm, LETI Pharma, Novartis, Regeneron, Sanofi, Stallergenes, Takeda. 

HR: Co-Founder STERNA Biologicals and SECARNA Pharmaceuticals. 

**Table 1.** Frequency of hypersensitivity reaction to biologics.

**Biologic**	**Target**	**Author**	**Year**	**HSR ** **%**	**IR ** **%**	**ISR ** **%**	**Urticaria ** **%**	**Anaphylaxis ** **%**
Adalimumab	TNF-α	Puxeddu et al. [243]	2012	3.5	–	1.5	1.5	0
		Tarkiainen et al. [244]	2015	18.1		17.0	–	–
		EMA [245]	2023	1.0 – 10.0		12.9	1.0 – 10.0	0.01 – 0.1
		FDA [246]	2023	6.0		5.0 – 20.0	6.0	–
Benralizumab	IL-5R	Castro et al. [247]	2014	–		16.0	–	–
		Park et al. [248]	2019	–		0	0 – 2.0	–
		Liu et al. [249]	2019	–		2.6–17.5	–	–
		FDA [250]	2019	3.0		2.2	3.0	3.0
		Bourdin et al. [251]	2019	0 – 3.2		3.2 – 6.5	–	–
		EMA [252]	2023	up to 10.0		2.2	up to 10.0	–
		Yamaguchi et al. [253]	2024	0.3		–	–	0.3
Brodalumab	IL-17RA	FDA [254]	2017	–		1.5	1.0	–
		Iznardo et al. [255]	2020	< 1.0		1.8	–	–
		Kim et al. [256]	2023	–		1.3	–	–
		EMA [257]	2023	–		1.0 – 10.0	–	0.01 – 0.1
Certolizumab	TNFα	FDA [258]	2022	–		1.7 – 3.2	–	–
		EMA [259]	2023	–		1.0 – 10.0	–	0.01 – 0.1
		Kim et al. [256]	2023	–		3.9	–	–
Dupilumab	IL-4Rα	Ou et al. [260]	2018	–		13.2	–	–
		Halling et al [261]	2021	–		5.3	–	–
		EMA [262]	2023	–		1.0 – 10.0	–	0.01 – 0.1
		Kim et al. [256]	2023	–		11.3	–	–
		FDA [263]	2024	< 1.0		6.0 – 38.0	< 1.0	< 1.0
		Simpson et al. [264]	2024	–		3.0	–	–
		Yew et al. [265]	2024	–		2.0	5.9	–
Etanercept	TNF-α-RII	Puxeddu et. al. [243]	2012	5.3	–	1.6	2.0	0.8
		Tarkiainen et al. [244]	2015	11.3		7.5	–	–
		Girolomoni et al. [266]	2018	–		10 – 49.0	–	–
		Codreanu et al. [267]	2019	–		0.8	–	0.8
		FDA [268]	2023	< 1.0		15 – 43.0	< 2.0	< 2.0
		EMA [269]	2024	–		13.6 – 36.0	0.1 – 1.0	0.01 – 0.1
Etokimab	IL-33	Chen et al. [270]	2019	–	–	25.0	16.7	–
		Chinthrajah et al. [271]	2019	–	–	26.7	6.7	0
		NCT03614923 - Eclipse [272]	2022	–	–	2.8 – 5.7	–	–
Fezakinumab	IL-22	–	–	–	–	–	–	–
Garadacimab	FXIIa	Craigh et al. [273]	2023	0	–	5.0	–	0
Guselkumab	IL-23	Langley et al. [274]	2018	0		1.1		0
		FDA [275]	2020	–		4.5	< 1.0	–
		European Comission [276]	2020	0.1 – 1.0		1.0 – 10.0	0.1 – 1.0	0.1 – 1.0
		Coates et al. [277]	2021	–		1.8	0.4	0
		McInnes et al. [278]	2022	–		2.5 – 2.7	-	0
		Danese et al. [279]	2024	0		4.0	-	0
Infliximab	TNF-α	Maggi et al. [280]	2011	–	1.0 – 27.0	–	–	–
		Puxeddu et al. [243]	2012	13.8	–	0	4.4	9.3
		Tarkiainen et al. [244]	2015	34.1	–	1.9	–	1.9
		Lichtenstein et al. [281]	2015	–	5.0 – 23.0	–	4.0	–
		Panés et al. [282]	2019	–	13.0	–	–	–
		FDA [283]	2023	–	< 20.0	–	< 1.0	< 1.0
		EMA [284]	2024	–	> 10.0	1.0 – 10.0	1.0 – 10.0	0.1 – 1.0
Ixekizumab	IL-17A	FDA [285]	2022	≤ 0.1		17.0	≤ 0.1	≤ 0.1
		EMA [286]	2023	–		> 10.0	0.1 – 1.0	0.01 – 0.1
		Kim et al. [256]	2023	–		11.2	–	–
		Mastorino et al. [287]	2023	–		3.1	–	–
		Ying et al. [288]	2023	0.3		3.0 – 9.7	3.3	–
Lanadelumab	Plasma kallikrein	FDA [289]	2018	1.0		45.0 – 57.0	–	–
		Craig et al. [290]	2021	–		5.1 – 62.5	–	–
		Hide et al. [291]	2023	–		50.0	–	–
		EMA [292]	2024	1.2		52.4	–	–
Lebrikizumab	IL-13	Hanania et al. [293]	2015	0 – 0.9		11.1 – 20.5	–	0 – 0.9
		Hanania et al. [294]	2016	–		6.0 – 10.0	–	< 1.0
		Simpson et al. [295]	2018	–		1.3	–	0
		Korenblat et al. [296]	2018	–		2.9	–	1.0
		Austin [297]	2020	0		7.0	–	0
		EMA [289]	2023	–		2.6	–	–
		Paller et al. [299]	2023	–		2.4	2.9	0
		Stein Gold et al. [300]	2023	0		2.6	–	0
Ligelizumab	Cε3 domain of IgE	Gauvreau et al. [301]	2016	–		12.5 – 25.0	0	0
		Maurer et al. [302]	2019	–		4.0 – 7.0	–	0
		Wood et al. [239]	2022	–		–	17.0	0.4
		Maurer et al. [303]	2024	6.0 – 11.0		4.0 – 11.0	–	< 1.0
Mepolizumab	IL-5	Pavord et al. [304]	2012	≤ 1.0	5.0–12.0	–	–	0
		Lugogo et al. [305]	2016	< 1.0	<1.0	3.0	–	0
		Leung et al. [306]	2017	0 – 1.0	5.0 – 12.0	3.0 – 9.0	4.0 – 16.0	0.002
		Khatri et al. [307]	2019	2.0	–	12.0	–	0
		Chapman et al. [308]	2019	< 1.0	–	3.0	< 1.0	0
		EMA [309]	2022	1.9 – 3.0	–	6.0 – 7.0	–	0
		FDA [310]	2023	1.0 – 4.0		2.0 – 15.0	–	–
		Ishii et al. [311]	2023	< 1.0		–	–	< 1.0
Nemolizumab	IL-31Rα	Nemoto et al. [312]	2016	–		–	–	0
		Kabashima et al. [313]	2018	–		2.0	2.0 – 6.0	–
		Silverberg et al. [314]	2020	–		1.8 – 3.5	–	–
		Ständer et al. [315]	2020	–		3.0	–	–
		Kabashima et al. [185]	2022	–		< 1.0	–	–
		Igarashi et al. [316]	2023	–		2.2	–	–
Omalizumab	IgE	Cox et al. [317]	2007	< 0.2		–	–	0.09
		Di Bona et al. [318]	2017	–		3.4	1.0	0
		FDA [319]^a^	2023	–		12.0 – 45.0	0.2	0.1
		FDA [319]^b^	2023	–		0.6 – 2.7	–	–
		EMA [320]	2023	–		2.7	0.1–1.0	0.2
		Kim et al. [256]	2023	–		4.5	–	–
Reslizumab	IL-5	Castro et al. [321]	2015	–	–	1.0 – 2.0	–	< 1.0
		Murphy et al. [322]	2017	< 1.0	< 1.0	< 1.0	< 1.0	0
		FDA [323]	2019	–	–	–	–	0.3
		Virschow et al. [324]	2020	–	–	–	–	< 1.0
		Bernstein et al. [325]	2020	0	–	6.0 – 11.0	–	–
		EMA [326]	2023	0.19	0.19	–	–	0.19
Rituximab	CD20	Terrier et al. [327]	2010	–	9.0	–	–	1.4
		Maggi et al. [280]	2011	–	10 – 77.0	–	–	–
		FDA (s.c.) [328]	2021	–	–	13–26.0	–	–
		FDA (i.v.) [329]	2021	–	≥ 25.0	–	2.0 – 8.0	< 2.0
		BCCA [330]	2024	1.0 – 10.0	14 – 77.0	20.0	7.0	–
		EMA [331]	2024	1.0 – 10.0	> 10.0	< 20.0	1.0 – 10.0	0.01 – 0.1
		Riveiro-Barciela et al. [332]	2024	–	9.0	–	–	2.8
Secukinumab	IL-17A	Blauvelt [333]	2016	–		0.7	–	–
		Deodhar et al. [334]	2019	2.4		0.8–1.3	–	–
		Grace et al. [335]	2020	–		25.0	–	–
		Asawanonda et al. [336]	2022	–		0.6	–	–
		Li et al. [337]	2022	–		2.3	–	–
		FDA [338]	2023	0.01 – 0.1		–	0.6 – 1.2	–
		EMA [339]	2023	–		–	0.1 – 1.0	0.01 – 0.1
		Kim et al. [256]	2023	–		1.9	–	–
Tezepelumab	Anti-TSLP	Menzies-Gow et al. [340]	2021	–		3.6	–	0
		Corren et al. [341]	2023	–		4.0	–	0
		EMA [342]	2024	–		3.8	–	–
Tralokinumab	IL-13	Wollenberg et al. [343]	2019	–		5.2	–	–
		Panettieri et al. [344]	2018	–		4.0–5.4	–	0
		Busse et al. [345]	2019	–		15.7	–	0
		Carlsson et al. [346]	2019	13.2 – 25.9		–	< 1.0	0
		Silverberg et al. [347]	2021			6.7		
		FDA [348]	2023	–		7.4 – 11.1	–	–
		EMA [349]	2023	–		7.2	–	–
		Paller et al. [40]	2023	–		2.1 – 9.2	–	0
Upadacitinib	JAK inhibitor	FDA [350]	2023	2.0 – 3.0		–	2.0 – 3.0	2.0 – 3.0
		EMA [351]	2023	0.1 – 1.0			1.0 – 10.0	0.1 – 1.0
Ustekinumab	IL-12 / IL-23	Ghosh et al. [352]	2019	<1.0	0.1	–	< 1.0	0
		FDA [353]	2023	0.08	–	1.0 – 5.0	–	0.1
		EMA [354]	2023	0.1 – 1.0	1.9	0.1 – 10.0	0.08	0.01 – 0.1
		Kim et al. [256]	2023	–	–	2.8	0.1 – 1.0	–

^a^Results of clinical studies with asthma in FDA 2023 label. ^b^Results of pooled chronic idiopathic urticaria trials in FDA 2023 label. JAK = Janus kinase; TSLP = thymic stromal lymphopoietin; HSR = hypersensitivity reaction; IR = infusion reaction, substance-specific; ISR = injection-site reaction.

**Table 2.** Laboratory tests before administration of immunosuppressive or immunomodulating drugs [1].

Infectious agents	Test
Hepatitis B virus	– anti-HBS quantified – HBs Antigen, anti-HBs and – anti-HBc
Hepatitis C virus	(anti-Hepatitis C)
Hepatitis A virus	(anti-HAV IgG)
Epstein-Barr virus	anti-EBV
Cytomegalovirus	anti-CMV IgG and IgM
Herpes virus	anti-HSV q and 2: IgG and IgM
Varicella zoster virus	Anti-VZ IgG
Syphilis	VDRL or TPPA

**Table 4.** Biologics investigated in ongoing studies for the indication of eosinophilic esophagitis.

**Name**	**Target**	**Phase**
Barzolvolimab	KIT-TKI	Phase II [228]
Etrasimod	Sphingosine 1P-R modulator	Phase II [229]
Cendakimab	IL-13 inhibitor	Phase III [230]
Tezepelumab	Anti-TSLP antibody	Phase III [231]

**Table 3.** Biologics investigated in ongoing studies for the indication of chronic spontaneous urticaria.

**Name**	**Target**	**Phase**
UB-221	IgER antagonists	Phase II [221]
Lirentelimab	Siglec-8 agonist	Phase II [222]
Barzolvolimab	KIT-TKI	Phase II [223]
Rilzabrutinib	Bruton-TKI	Phase II [224]
TAS-5315	Bruton-TKI	Phase II [225]
